# The role of PKCζ in cord blood T-cell maturation towards Th1 cytokine profile and its epigenetic regulation by fish oil

**DOI:** 10.1042/BSR20160485

**Published:** 2017-03-27

**Authors:** Hani Harb, James Irvine, Manori Amarasekera, Charles S. Hii, Dörthe A. Kesper, YueFang Ma, Nina D’Vaz, Harald Renz, Daniel P. Potaczek, Susan L. Prescott, Antonio Ferrante

**Affiliations:** 1 Institute of Laboratory Medicine and Pathobiochemistry, Molecular Diagnostics, Philipps University Marburg, Marburg, Germany; 2 Department of Immunopathology, SA Pathology, Women and Children’s Hospital Campus, School of Medicine; Robinson Research Institute and School of Biological Science, University of Adelaide, Adelaide, South Australia, Australia; 3 School of Paediatrics and Child Health, University of Western Australia, Perth, Western Australia, Australia; 4 John Paul II Hospital, Krakow, Poland

**Keywords:** accessory cells, cord blood T-cell maturation, epigenetics, fish oil, neonates, PKCζ

## Abstract

While immunodeficiency of immaturity of the neonate has been considered important as the basis for unusual susceptibility to infection, it has also been recognized that the ability to progress from an immature Th2 cytokine predominance to a Th1 profile has relevance in determining whether children will develop allergy, providing an opportunity for epigenetic regulation through environmental pressures. However, this notion remains relatively unexplored. Here, we present evidence that there are two major control points to explain the immunodeficiency in cord blood (CB) T-cells, a deficiency in interleukin (IL)-12 (IL-12) producing and IL-10 overproducing accessory cells, leading to a decreased interferon γ (IFNγ) synthesis and the other, an intrinsic defect in T-cell protein kinase C (PKC) ζ (PKCζ) expression. An important finding was that human CB T-cells rendered deficient in PKCζ, by shRNA knockdown, develop into low tumour necrosis factor α (TNFα) and IFNγ but increased IL-13 producing cells. Interestingly, we found that the increase in PKCζ levels in CB T-cells caused by prenatal supplementation with fish oil correlated with modifications of histone acetylation at the PKCζ gene (*PRKCZ*) promoter. The data demonstrate that PKCζ expression regulates the maturation of neonatal T-cells into specific functional phenotypes and that environmental influences may work via PKCζ to regulate these phenotypes and disease susceptibility.

## Introduction

The distinct functional characteristics of neonatal T-cells compared with those of adults are now understood to reflect an adaptive immune profile in response to the unique developmental context of intrauterine life [1–10]. Rather than in a simple linear manner from immaturity to maturity, the immune system appears to develop in distinct functional waves that serve different purposes at different developmental stages [3,4,11–14]. Neonatal T-cell responses are generally characterized by diminished interferon γ (IFNγ) production and bias towards T-helper cells (Th) type 2 (Th2) cytokines compared with adults [5,15–17]. In the postnatal period, T-cells undergo a series of age-related changes to eventually achieve the regulated Th1-dominant ‘mature’ patterns of response observed in adult life [15–19]. Early differences in T-cell function, evident both at birth and in trajectory of postnatal maturation, have been shown to precede the development of allergic inflammation [18,20,21].

Previous work in our laboratory revealed that neonates have reduced capacity to activate certain protein kinase C (PKC)-dependent mitogen-activated protein kinases (MAPKs) upon TCR/CD28 stimulation compared with adults [22]. MAPKs play a crucial role in the regulation of cytokine production in T-cells in a PKC-dependent manner, for which specific PKC isozymes are required for different signalling pathways [23–25]. The lower capacity of neonatal T-cells to activate MAPKs has been attributed to lower levels of PKC isozyme expression leading to a relatively weak signalling through PKC [22]. Of the PKC isozymes, PKC ζ (PKCζ), an atypical isozyme of PKC family, is important for regulating asymmetric T-cell division that determines the subsequent fate of the cells [26]. We have previously reported that expression of PKCζ by neonatal T-cells was relatively lower as compared with adults and its levels in cord blood (CB) T-cells predicted the development and severity of allergic disease, indicating the significance of CB PKCζ levels as a predictive marker of disease risk [27,28]. Furthermore, the level of T-cell PKCζ at birth was positively associated with the capacity for IFNγ and tumour necrosis factor α (TNFα) production by *in vitro* matured neonatal T-cells and was negatively associated with allergen-specific interleukin (IL)-13 (IL-13) production at 6 months of age [28] suggesting that PKCζ may be involved in driving age-related maturation of T-cell response pattern. Furthermore, our previous studies demonstrate that maternal fish oil (ω-3 fatty acids) supplementation causes both immunomodulation and allergy protection in the offspring [29] and alters PKCζ expression by CB T-cells [27], suggesting that the genomic region that encodes PKCζ is readily amenable to modulation by *in utero* nutritional exposures.

Despite these developments in neonatal immunology, the basis for the physiological immunodeficiency of immaturity, as well as factors regulating the development of Th1 profiles, remain ill defined. Here, we demonstrate that the major defect in CB mononuclear cells (MCs) (CBMCs) in producing Th1 cytokines lies in not only in an inability of the accessory cells to produce IL-12, associated with an elevated production of IL-10, but also in an intrinsic T-cell maturation defect that is regulated by PKCζ to develop into Th1 cytokine producers. Interestingly, the data suggest that the increase in PKCζ expression following prenatal supplementation with fish oil is likely to be epigenetically controlled.

## Materials and methods

### Preparation of MCs and T-cells

Human CB or peripheral blood (PB) for MCs isolation was obtained according to the institution’s guidelines on human ethics from healthy neonates who had no complications at the delivery or from healthy adult volunteers. MCs were isolated from CB and PB as previously described [27]. T-cells were purified by removing adherent monocytes in plastic tissue-culture dishes and filtering the non-adherent lymphocyte fraction through two cycles of nylon wool columns using an established protocol [27]. The T-cell preparation was ≥95% pure and >99% viable as determined by FACS analysis and Trypan Blue dye exclusion assay respectively. Purified CD4^+^ T-cells were isolated from CBMCs as previously described [17].

### Preparation of cell lysate

CB T-cells were lysed in 100 μl of cold lysis buffer [20 mM Hepes, pH 7.4, 0.5% NP40 (v/v), 100 mM NaCl, 1 mM EDTA, 2 mM Na_3_VO_4_, 2 mM DTT, 1 mM PMSF, 2 mM *p*-nitrophenylphosphate and 10 mg/ml each of leupeptin, aprotinin, pepstatin A and benzamidine] for 2 h (4°C) with constant mixing. The samples were centrifuged (12000 g for 30 s) and the protein content of the supernatants was determined by the Lowry’s protein estimation method. Samples were stored at -20°C until Western blotted.

### Measurement of PKCζ levels

PKCζ expression in neonatal T-cells was analysed by Western blot assay as described previously [22,28]. Briefly, proteins were separated by SDS/PAGE (12% gel) and transferred to nitrocellulose membrane (Bio-Rad Laboratories, Gladesville, NSW, Australia). The membranes were stained with Ponceau S to assess the evenness of transfer between the lanes. Following blocking, the membrane was probed with mouse monoclonal anti-PKCζ antibody (Santa Cruz Biotechnology, Dallas, TX, U.S.A.), washed and then incubated with HRP-conjugated sheep anti-mouse antibody. The immune complexes were detected by ECL using Western Lightning Plus-ECL (Perkin Elmer, Melbourne, VIC, Australia).

### LPS-induced cytokine production

Human CBMCs and PB MCs (PBMCs) were incubated with lipopolysaccharide (LPS) for 48 h at 37°C before supernatants were collected. Under the same time and temperature conditions, CBMCs were also cultured in the presence of either anti-IL-10 (IL-10 neutralizing) antibody or IL-12 alone or with LPS. Cytokine levels were measured in supernatants using Cytometric Bead Array Flex Sets and analysed on BD FACS Array Bioanalyzer (BD Biosciences, Franklin Lakes, NJ, U.S.A.) [30].

### Knockdown of PKCζ in CB T-cells

PKCζ knockdown in human CB T-cells was carried out essentially as described recently for human macrophages [31]. Predesigned shRNA specific for PKCζ and non-targeting control shRNA were purchased from Sigma–Aldrich (Castle Hill, NSW, Australia). The human T-cell Nucleofection kit was from Amaxa (Lonza, Wakersville, MD, U.S.A.). Approximately 10^6^ cells were added to 4 μg of non-targeting control shRNA or PKCζ-specific shRNA in cuvettes and the cells were transfected using programme Y-010 according to the manufacturer’s instructions. After transfection, T-cells were cultured for 24 h before harvesting for maturation studies. An aliquot of the cultures was used to confirm the knockdown of PKCζ by Western blot analysis. Cell viability monitored by the Trypan Blue dye exclusion assay was >90%, being consistent with the information provided by the Nucleofection kit.

### Neonatal T-cell maturation assay

Human CB T-cells, including those which were deficient in PKCζ, were maturated as previously described using phytohaemagglutinin (PHA) and IL-2 [22,28]. Maturation was gauged by flow cytometry, measuring the expression of CD45RA and CD45RO using anti-CD45RA-APC, anti-CD45RO-PE, anti-CD3-FITC or isotype control mix (IgG2b-APC, IgG2a-PE and IgG1-FITC) antibodies (all BD Biosciences). The data were analysed on a BD FACScan using BD CellQuest software (BD Biosciences). Cultures were set up such that at each time point, a count was made and cell concentrations were re-adjusted based on the number of viable cells as described under ‘Results’ section.

### Responses of *in vitro* matured CB T-cells

Immune responses of *in vitro* maturated human neonatal T-cells were induced by adding PHA and PMA and measuring lymphocyte proliferation by quantifying the uptake of tritiated thymidine (^3^H-TdR) and by the cytokine release in 72-h cultures [30].

### Analysis of H3 and H4 histone acetylation levels in CB CD4^+^ T-cells

A neonatal cohort for the analysis of the histone acetylation derived from a previously conducted clinical trial, in which mothers were daily supplemented with either fish oil or placebo from 20 weeks of gestation until delivery [8]. CD4^+^ T-cells were obtained from 70 neonates (placebo, *n*=34; fish oil, *n=*36). Histone acetylation levels at promoter of PKCζ gene (*PRKCZ*) and, additionally, other loci involved in T-cell polarization, such as those encoding IL-4, -5, -9 and -13 (*IL4*, *IL5*, *IL9* and *IL13* respectively), T-box 21 (*TBX21*), IFNγ (*IFNG*), GATA-binding protein 3 (*GATA3*) and forkhead box P3 (*FOXP3*), were measured using a previously validated chromatin immunoprecipitation (ChIP) assay [32]. For the analysis, the percent enrichment of the negative control (IgG) was subtracted from this value and then normalized to that of *RPL32*, a positive control gene. The advantage of the application of a positive control gene measurement is that the same ChIP samples are used to analyse both, target and control sequences, thus minimizing variation caused by sample handling. The normalization was done according to the following formula [33,34]:

$$\text{Relative enrichment of desired gene}=\frac{\%\;\text{Enrichment of desired gene}}{\%\;\text{Enrichment of }RPL32}$$

### Statistical analysis

Data are presented as mean ± S.E.M. Statistical comparisons were performed using the unpaired *t* test or the ANOVA followed by Bonferroni’s multiple comparison test, as appropriate. Since the data obtained in the epigenetic analysis did not demonstrate a normal distribution when analysed with Shapiro–Wilk W test, they were subjected to square root transformation before entering statistical comparisons.

## Results

### Deficient production of IFNγ by CB T-cells is a function of abnormal synthesis of IL-12 and IL-10 by accessory cells

While the prime focus of this work was to examine the role of PKCζ in CB T-cell development-specific functional phenotype, studies on IL-12 and IL-10 were conducted to provide a comparison with the T-cell deficiency *per se*. Whereas the production and role of IL-12 has been previously reported, findings on IL-10 production are controversial and indeed contradictory to that published previously by our group [35–38]. Furthermore, the role of altered IL-10 production in CB T-cell IFNγ production has not been examined. It has been shown that LPS-induced T-cell stimulation and production of IFNγ is dependent on accessory cells i.e. antigen presenting cells for co-stimulation [39]. This was the model used in our study.

**Figure 1:**
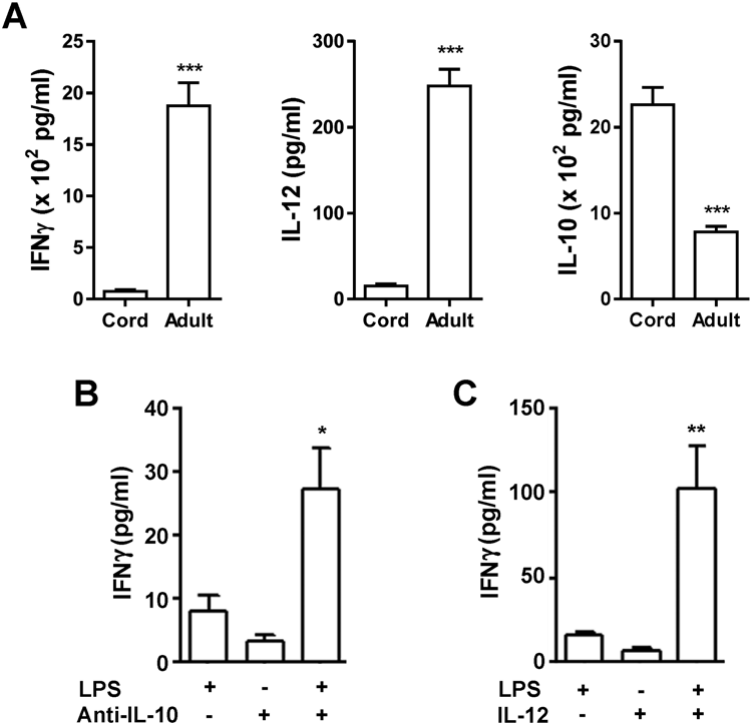
Deficient production of IFNγ by human CBMCs is a function of altered cytokine milieu (**A**) Reduced production of IFNγ and IL-12 but increased synthesis of IL-10 by human CBMCs compared with adult PBMCs was observed in response to LPS (30 ng/ml). (**B**) Neutralization of IL-10 with an anti-IL-10 monoclonal antibody (10 μg/ml) resulted in a significant increase in IFNγ production by LPS-stimulated (30 ng/ml) CBMCs. (**C**) Addition of exogenous recombinant human IL-12 (2 μg/ml) increased the IFNγ synthesis by LPS-stimulated CBMCs. Data are expressed as mean ± S.E.M of three experiments. Statistics: *, *P*<0.05; **, *P*<0.01; ***, *P*<0.001.

The production of IFNγ by human CBMCs was of the order of 5% of that observed with PBMCs from adults when accessory cells were stimulated with LPS (Figure 1A, left panel). This decrease in IFNγ production was not surprising since the accessory cells showed significantly lower production of IL-12 (Figure 1A, middle panel) and indeed significantly more IL-10 (Figure 1A, right panel), most likely to be responsible for the decreased IFNγ synthesis. Culturing LPS-stimulated CBMCs in the presence of an anti-IL-10 neutralizing antibody resulted in an increase in IFNγ production (Figure 1B). The addition of exogenous IL-12 to LPS-induced CMBCs overcame the inability of the cells to produce IFNγ (Figure 1C). These data reveal that the reduced CB T-cell response is at least partly a function of the altered extrinsic cytokine milieu of accessory cells.

### PKCζ regulates the functional phenotype potential of maturating CB T-cells

Previously, we found that low expression of PKCζ in CB T-cells resulted in mature T-cells which had reduced TNFα and IFNγ production [28]. This has been further studied now in CB T-cells that had been made PKCζ-deficient using shRNA.

**Figure 2:**
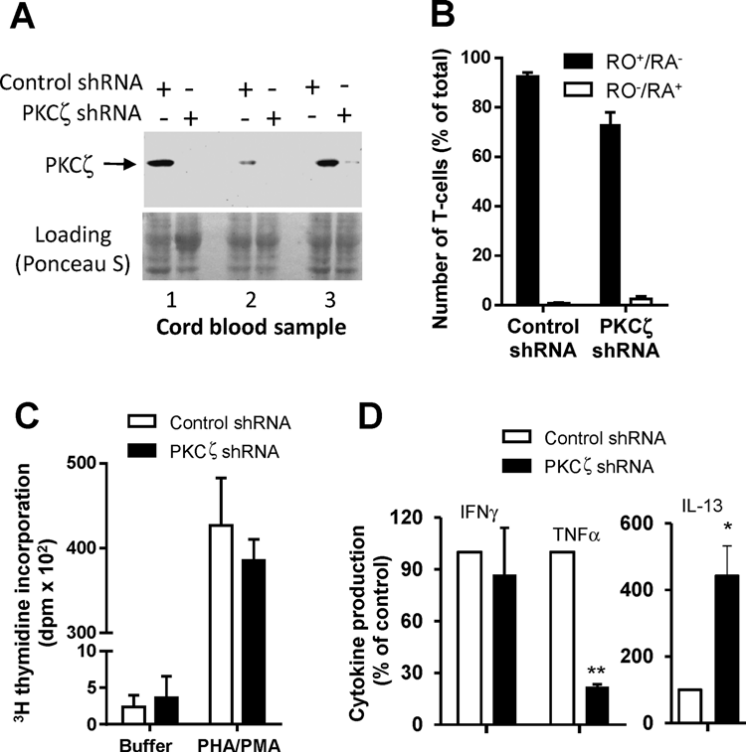
PKCζ regulates T-cell functional phenotype potential during maturation of human CB T-cells in culture Human CB T-cells were maturated to CD45RO^+^/RA^–^ phenotype by culturing with addition of PHA (day 1) and IL-2 (day 3) over 7 days. (**A**) PKCζ knockdown was achieved by nucleofection of CB T-cells with specific shRNA. Nucleofection with PKCζ shRNA led to the loss of PKCζ in T-cells, as demonstrated by Western blot of three separate CB samples. (**B**) Evaluating day 8 of cultures for the level of T-cell maturation by the levels of expression of CD45RA/CD45RO. (**C**,**D**) T-cells developed on PKCζ-deficient background and then stimulated with PHA/PMA were examined for (C) proliferation and (D) cytokine (TNFα, IFNγ and IL-13) production. The effects of PKCζ deficiency on cytokine synthesis are expressed as % of control production where the control values were 460 ± 115.5 pg/ml, 350.5 ± 125 pg/ml and 96 ± 57 pg/ml respectively (D). Results are expressed as mean ± S.E.M. of three experiments. Statistics: *, *P*<0.05; **, *P*<0.01.

CB T-cells were nucleofected with PKCζ-specific shRNA or control shRNA on day 0. Examination by Western blotting at 24 h following nucleofection demonstrated successful knock down of PKCζ protein (Figure 2A). Cell counts, scoring only live Trypan Blue-negative cells, were determined and the cells were then cultured at 1 × 10^6^ cells/ml in the presence of the maturation-promoting agents, PHA added at 24 h following nucleofection (day 1) and IL-2 on day 3. Viable cells were enumerated on days 3, 6 and 8 and the cells were reseeded (1 × 10^6^/ml) on days 3 and 6, with the re-addition of PHA and IL-2. As expected, cell number increased in response to IL-2. No significant differences in cell counts were evident between the control and PKCζ-depleted cells on all days except for day 6. At this time point, the increase in cell number was less in the PKCζ-depleted group than in the control group; control and PKCζ shRNA-nucleofected cultures yielded 2.59 ± 0.16 × 10^6^ and 1.34 ± 0.14 × 10^6^ live cells/ml, respectively (*P*<0.05, *n*=3). The resultant cells in cultures were all mature as assessed by the level of CD45RO expression on day 8 (Figure 2B; Supplementary Figure S1). The matured cells were also then used to determine whether CB T-cells maturing in the presence or absence of PKCζ exhibited different functional responses. Direct stimulation of purified T-cells was achieved by adding PHA/PMA. Both T-cell populations responded similarly in relation to lymphoproliferation (Figure 2C). However when they were examined for cytokine production, the T-cells resulting from the PKCζ-deficient group produced substantially lower amounts of TNFα and lower amounts of IFNγ but increased concentrations of IL-13 (Figure 2D). These findings suggest that PKCζ plays an important role in the development of Th1 functional phenotype during T-cell maturation and that the reduced Th1 capacity observed in neonates is underscored by lower PKCζ levels.

### Prenatal fish oil supplementation is associated with higher transcriptional abilities of the PKCζ-encoding gene *PRKCZ*

Based on the increasing evidence that *in utero* nutritional exposures have the capacity to epigenetically modulate specific genomic regions in the offspring [40], we speculated that maternal fish oil intake may modify epigenetic marks at *PRKCZ* locus. As our previous epigenome-wide DNA methylation analysis of neonatal CD4^+^ T-cells did not implicate changes in DNA methylation in fish oil-induced PKCζ up-regulation [41], we hypothesized that these effects are more likely to be mediated by other epigenetic or post-transcriptional effects that modulate cellular function. To this end, we compared the H3 and H4 histone acetylation profiles in CD4^+^ T-cells obtained from neonates whose mothers were supplemented with either fish oil or placebo during pregnancy.

**Figure 3:**
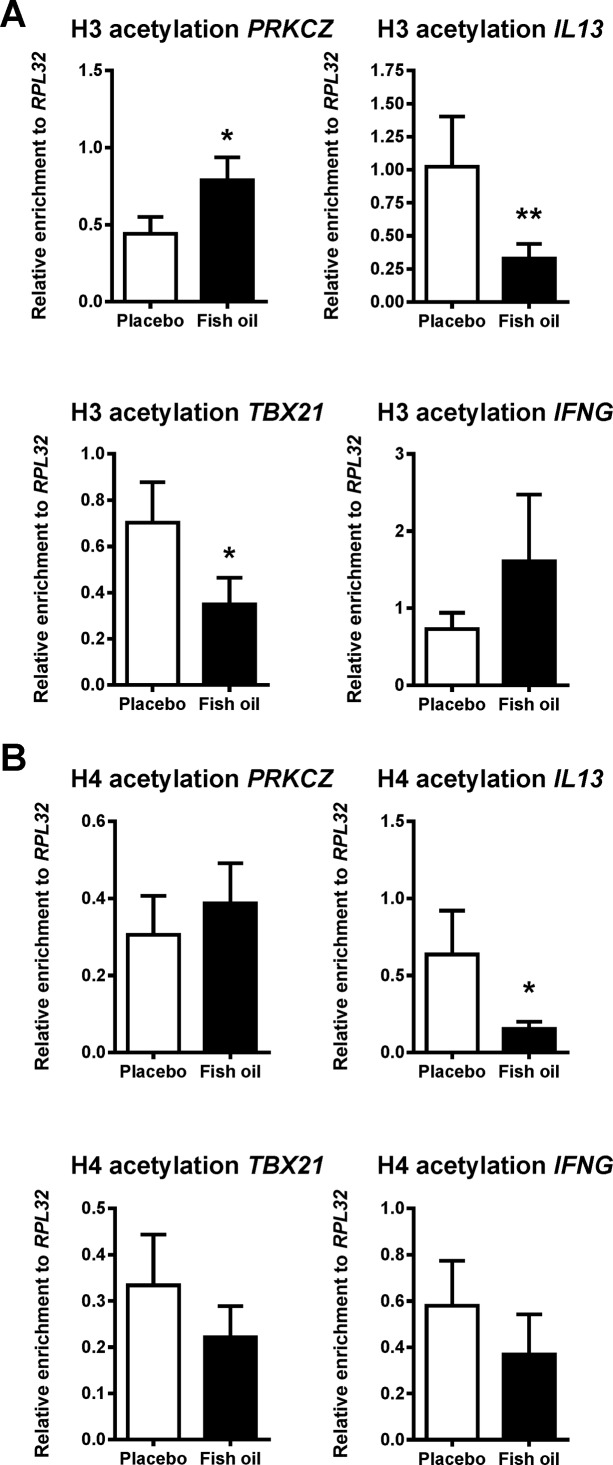
PKCζ gene (*PRKCZ*) promoter histone acetylation levels in CB CD4^+^ T-cells differ between offspring of mothers treated during pregnancy with fish oil compared with placebo CB CD4^+^ T-cells were obtained from 70 neonates (placebo, *n*=34; fish oil, *n*=36) and ChIP assayed for histone acetylation at promoters of genes encoding PKCζ (*PRKCZ*), IL-13 (*IL13*), T-box 21 (*TBX21*) and IFNγ (*IFNG*). (**A**) When compared with placebo, acetylation of histone H3 in fish oil group was higher in case of *PRKCZ* promoter and lower in corresponding regions of *IL13* and *TBX21*. (**B**) Histone H4 acetylation at *IL13* promoter was lower in fish oil arm of the study than in controls. Results, expressed as relative enrichment (i.e. after normalization to *RPL32*), are given as mean ± S.E.M. Statistics: *, *P*<0.05; **, *P*<0.01.

We found that in CD4^+^ T-cells obtained from CB of babies born from mothers treated during pregnancy with fish oil, a higher acetylation of histone H3, corresponding to a more transcriptionally permissive chromatin status [32,42–46], was observed at the promoter region of *PRKCZ*, the gene encoding PKCζ (Figure 3A). Additionally, in a simultaneous analysis of other loci involved in T-cell polarization (Figure 3A,B, Supplementary Figures S2A,B), we found that the acetylation levels of either H3/H4 or H3 alone at promoters of *IL13* or *TBX21*, respectively, were lower in the fish oil group compared with placebo. Thus, fish oil supplementation during pregnancy is associated with epigenetic regulation of PKCζ and other T-cell-related loci.

## Discussion

The data demonstrate that the T-cell responses in terms of cytokine production are reduced in CB cells because the accessory cells produce significantly less IL-12, confirming previously reported findings [47] and increased amounts of IL-10, consistent with our reported data [38]. Supplementing the cells with IL-12, as reported previously [47] enabled the CB T-cells to significantly increase their production of IFNγ. We now report that the addition of anti-IL-10 antibody also increases the production of IFNγ. While this argues against an intrinsic T-cell defect, it is best explained by the fact that the action of IL-12 is via predominantly the JAK2/Tyk2-STAT1/3/4 pathway and bypasses the defective early signalling such as p56lck, ZAP70, phospholipase C, PKC and MAPKs [22,48], required when activation is induced via the TCR–CD3 complex. While MAPKs function normally in CB T-cells, PKC and p56lck are deficient [22,48].

We have previously shown that CB T-cells have deficient expression of PKCζ and other PKC isozymes [22,27,28] but that these normalize within 16 h after inducing maturation in the *in vitro* maturation model [22]. Thus, by day 8 when the T-cells are fully matured, the PKC signalling pathway can be engaged to stimulate T-cell functions. Since we have previously found that PKCζ levels in CB T-cells correlate with their maturation towards IFNγ-, TNFα-producing T-cells [28], it is likely that PKCζ regulates the functional phenotype of the matured T-cells. Importantly, our present data show that knocking down PKCζ in CB T-cells altered their functional phenotype following maturation in culture. The cells had reduced ability to produce TNFα and IFNγ but increased IL-13 following stimulation with PHA/PMA. This suggests that PKCζ is likely to be important for the development of Th1 cytokine producing cells. At this stage, it is not clear as to why there was a preferential role for PKCζ in the development of TNFα compared with IFNγ producing T-cells but this identifies further avenues to be explored in trying to understand T-cell maturation in neonates.

It is tempting to speculate that the levels of PKCζ dictate the selection of cells that may be skewed towards either Th1 or Th2 functional phenotype based on regulating their survival. A role for PKCζ in regulating asymmetric T-cell division has been reported [26]. This process, characterized by the unequal apportioning of cellular contents of the parent T-cell into the two daughter cells, enables lymphocyte fates to diverge early in an immune response when T-cells are first stimulated by accessory cells [49,50]. Whether our observation represents a new role for PKCζ or is related to asymmetric division requires further investigation. In addition, while, we have emphasized the relationship between PKCζ levels and allergy development risk, it is evident that the concept would encompass the development of autoimmunity through a positive or negative skewing towards Th1. This is also an area to be given future attention and may lead to benefits in treating or preventing these chronic inflammatory diseases.

It has been shown that children born from mothers supplemented during pregnancy with fish oil are at a lower risk of allergic disease development [27,29] and that their CB T-cells express higher PKCζ levels [27]. This suggests that PKCζ expression is amenable to prenatal nutritional exposures, possibly through epigenetic modifications such as DNA methylation or histone acetylation [51–53]. However, epigenome-wide analysis of neonatal CD4^+^ T-cells revealed that* in utero* exposure to fish oil did not significantly affect T-cell DNA methylation profiles [41]. In the present study, CB CD4^+^ T-cells obtained from offspring of fish oil-treated mothers showed higher acetylation levels of H3 histone at the promoter of *PRKCZ*, the PKCζ-encoding gene, corresponding to more transcriptionally permissive chromatin status and thus higher PKCζ synthesis [32,42–46]. Thus, the effects of prenatal fish oil supplementation [27,54] seem to be at least partly mediated through epigenetic control of PKCζ synthesis. One might also speculate that histone acetylation at PKCζ promoter represents a more common mechanism regulating PKCζ synthesis in maturating T-cells.

The window of opportunity to educate the immune system and reduce the risk of developing atopic conditions appears to be *in utero* and perhaps in the early postnatal life of an individual. As reported previously by us [27], fish oil supplementation *in utero* was associated with enhanced expression of PKCζ in the CB T-cells, perhaps providing an explanation for the protection against allergic responses. Although the validity of the hygiene hypothesis has recently been called into question [55], our data may also contribute to the understanding of the basis for the protection from allergic diseases that is associated with microbial exposure early in life [56,57]. We have previously reported that polyclonal stimulation of CB T-cells with PHA increased the level of PKCζ [22]. Thus, such early exposure and a subsequent increase in PKCζ expression, particularly in infants with low PKCζ or those with a family history of atopic diseases, could promote a Th1/Th2 balance skewed towards Th1 that favours a non-atopic immune system. In an evolutionary context, the hunter gatherers had a favourable ω-3:ω-6 fatty acid ratio compared with what we have now and based on our data, we have evolved into a less-mature Th cell cytokine profile conducive with an increased incidence of allergic diseases.

In summary, our data point to the lack of synthesis of IL-12 and overproduction of IL-10 by accessory cells as a key defect leading to poor production of Th1 cytokines. Presumably, this is a mechanism of protecting the foetus. PKCζ, an atypical PKC isozyme pivotal for T-cell asymmetric division, has been identified as a very interesting candidate to underlie relative Th1 deficiency/Th2 bias of neonatal T-cells. In brief, PKCζ expression has been found to be relatively lower in neonatal T-cells when compared with their adult counterparts [22,28]. Furthermore, on the inter-individual level, higher PKCζ expression has been shown to correlate with the capacity of neonatal T-cells to produce more IFNγ upon stimulation and, at the same time, to be associated with a lower risk of allergic disease development in early childhood years [27,28]. The data obtained in the present study, using shRNA-mediated knockdown, demonstrate that while the ability of neonatal T-cells to proliferate is not regulated by PKCζ, cytokine secretion is. This provides an opportunity to dissect the requirements for controlling T-cell development and maturation from a Th2 to Th1 functional cytokine phenotype [58 ]. Indeed, it may provide a window of opportunity for intervention and reduce the risk of developing serious diseases such as allergy, especially, since we found that these levels of CB T-cell PKCζ could be altered by fish oil supplementation, in an epigenetic manner, which might actually be a more general mechanism regulating PKCζ levels and thus T-cell maturation.

## Abbreviations

CB: cord blood
CBMC: CB mononuclear cell
ChIP: chromatin immunoprecipitation
IFNγ: interferon γ
IL: interleukin
LPS: lipopolysaccharide
MAPK: mitogen-activated protein kinase
MC: mononuclear cell
PB: peripheral blood
PBMC: peripheral blood mononuclear cell
PHA: phytohaemagglutinin
PKC: protein kinase C
PKCζ: protein kinase C ζ
TBX21: T-box 21
Th (Th1, Th2): T-helper cells (type 1, type 2)
TNFα: tumour necrosis factor α
